# Editorial: Novel aspects of the immunological and structural barrier of the epidermis

**DOI:** 10.3389/fmolb.2023.1324920

**Published:** 2023-11-01

**Authors:** Ryan O’Shaughnessy, John Common, Danuta Gutowska-Owsiak

**Affiliations:** ^1^ Queen Mary University of London, London, United Kingdom; ^2^ Skin Research Institute of Singapore, Agency for Science, Technology and Research (A*STAR), Singapore, Singapore; ^3^ University of Gdańsk, Intercollegiate Faculty of Biotechnology of University of Gdańsk and Medical University of Gdańsk, Gdańsk, Poland

**Keywords:** epidermis, skin barrier, epidermal barrier, microbiome, skin disease

The importance of the epidermal barrier as a multifaceted mechanistic unit supporting whole-body homeostasis continues to grow in prominence. The spotlight again points towards this vital barrier tissue with the recent realization of significant importance for novel aspects in preventing skin diseases and skin infections, that have been tightly associated with atopic dermatitis (AD) and psoriasis. Unchecked, percutaneous defects progress to atopic diathesis and linked systemic manifestations. To this end, the skin provides interlinked mechanical, immunological, and antimicrobial protection, formed through keratinocyte differentiation, generating a multi-layered tissue with a structural and protective function. In addition, multiple cell types including keratinocytes, melanocytes, and immune cells crosstalk within the skin to create the first line of protection against pathogens and environmental threats, through the production of immunological mediators, recruitment of humoral and cellular immunity as well as the hosting of a commensal microbiome. The goal of this Research Topic was to bring together the latest advances and insights of epidermal biology, emphasizing both its immunological and physical aspects, providing a platform to present novel findings and summarise current knowledge, from both basic science and the clinical perspective. To this end, ten publications were accepted covering a broad range of research areas for epidermal barrier function in the form of 4 original articles and 6 reviews.

Multiple functions related to effective epidermal homeostasis has been implicated in various skin diseases. Protein and lipid-related barrier functions are associated with common, complex disorders such as atopic dermatitis (AD) but also many rarer and monogenic disorders with gene defects that result in ichthyosis or scaling phenotypes of the epidermis. One such condition is recessive X-linked ichthyosis. This disorder is caused by genetic defects in steroid sulfatase (*STS*) gene resulting in a cutaneous phenotype but also additional syndromic consequences. The mechanisms related to X-linked ichthyosis are still unclear and therefore McGeoghan et al. investigated the transcriptomic and lipidomic profiles related to *STS* gene deficiency. Findings included major alterations in epidermal differentiation and lipid metabolism as well as differentially expressed genes (DEGs) related to corneal transparency and behavioural disorders that are syndromic with the cutaneous phenotype. This work provides novel pathomechanistic data that will be important for future studies on X-linked ichthyosis.

Pemphigus represents a group of rare disorders with severe autoimmune blistering of the skin and mucosa. Autoantibodies are produced in pemphigus that target desmosomal adhesion proteins such as desmoglein 3 and desmoglein 1. The desmosome is a major junctional component of the skin responsible for maintaining tissue integrity and acting as an intracellular signalling platform within the epidermis. Lim et al., review the current literature around pemphigus discussing predisposing factors, underlying triggers for autoantibody production, pathogenic mechanisms, and the current emerging therapeutics. The review also provides an update on clinical phenotypes as well as detailed description of the cellular contribution related to T cells and myeloid cells pathogenesis.

Atopic dermatitis (AD) is a chronic inflammatory skin disease with an itchy erythematous rash. The pathomechanism of AD is complex with major contributions from the skin barrier, inflammation and the exposome. The complex genetics of AD has revealed over 50 potential loci that are associated with the phenotype of which the strongest genetic risk factor is *filaggrin* (*FLG*) gene, a skin barrier protein expressed in the upper layers of the epidermis. There is still much work to be done to further investigate DEGs in inflammatory skin diseases such as AD and Li et al., have taken a bioinformatics approach to this challenge. To identify potential effective diagnostic biomarkers in AD the team investigated gene expression from the Gene Expression Omnibus (GEO) database using comprehensive bioinformatics analysis. They identified biomarkers that were subsequently validated with immunohistochemistry showing CCR7, CXCL10, IRF7, MMP1, and RRM2 as potential biomarkers with diagnostic value. Finally, CIBERSORT algorithm was used to evaluate immune cell infiltration with CCR7 correlating with high levels of CD4^+^ naïve T cells.

Continuing the theme on skin inflammation, Dainichi et al., and co-workers have identified an orphan receptor, G protein-coupled receptor 15 ligand (GPR15L), encoded by *C10Orf99* gene as an important protein increased in frequently occurring skin diseases atopic dermatitis (eczema) and psoriasis. Over-expression of GPR15L reduces the expression of cornified envelope and keratinocyte differentiation markers keratin 10, filaggrin and loricrin, and increased the inflammatory response. Gene expression analyses revealed that GPR15L upregulated pathways and cognate transcription factors involved in signal transduction and stress inducible transcription, while reducing lipid metabolism, all responses that have previous been associated with skin barrier dysfunction.

The study by Kobiela et al., has reported alteration of cellular communication originating from epidermal keratinocytes, as the effect of allergic milieu and AD-relevant skin pathogens. Specifically, the study determined that the combined presence of AD cytokines and a common AD pathogen, *C. albicans*, resulted in an increased propensity of small extracellular vesicles (sEVs), a vehicle of long-distance communication, secreted by keratinocytes to interact with immunosuppressive Siglec receptors. The study also implicated the upregulation of specific enzymes involved in cellular glycosylation, i.e., β-Galactoside α-2,6-Sialyltransferase 1 (ST6GAL1) and Core 1 β,3-Galactosyltransferase 1 (C1GALT1) in remodelling of sEV surface glycans to allow for this keratinocyte related signalling effect to take place.

The theme of the microbiome is further addressed in the review by Baquero et al., who discussed the dynamic interaction between the epidermis and microbes and involving mechanisms of innate cell control. The authors explained how the threshold control is executed over overgrown pathogenic populations while allowing commensals to re-establish the original population density. The review further discussed the role of the epidermis in pathogen transmission and the factors behind interindividual variability in transmission efficiency.

The manuscript by Visscher et al., discusses the formation of the epidermal barrier from the foetal stage through birth and into adolescence, encompassing detailed information on the process of barrier maturation. The authors highlight the remodelling of the barrier proteome and lipidome as well as the importance of the vernix at the various life stages, and also touch on the role of the skin microbiome evolution past birth. The review by Çetinarslan et al. and co-workers provide an update on the contribution of microbiome imbalance, or “dysbiosis”, in atopic dermatitis (AD). This covers the complex interplay between innate immunity, lipid metabolism and dysbiosis, discussing the microbiome beyond the role of *Staphylococcus aureus*.

Arginase 1 is a key modulator of the skin microbiome but also regulates various mechanisms related to wound healing. In the review by Szondi et al. and co-workers, the multiple roles of Arginase 1 are discussed, including the well-characterised role in macrophage polarity, and also pro- and anti-inflammatory responses via innate immunity modulation. The review is concluded by describing how defects in Arginase 1 or Arginase 1 function can alter the microbiome of wounds contributing to chronic and non-healing wounds.

Another key component of innate immunity is the IL-1 family of cytokines. The review by MacLeod et al., provides a comprehensive description of the multiple functions of the IL-1 family in detecting external threats, controlling physical barrier function and inflammation. The authors also describe the deleterious effects on skin immunity when the IL-1 signalling pathways are defective. Finally, the potential role the IL-1 family has for translational therapeutics is reviewed with perspectives for future developments.

In conclusion, the articles in this Research Topic present original findings and aggregate prior knowledge on the novel aspects of the epidermal barrier. Appreciation of the importance of the epidermal barrier in physiology and pathology provides an important basis for development of new therapeutic strategies to address the diverse patient groups suffering from epidermal-associated skin diseases and their linked co-morbidities.

